# Supplementary material for: HSP90 inhibitors reduce cholesterol storage in Niemann-Pick type C1 mutant fibroblasts
Source: J Lipid Res. 2021 Sep 3;62:100114. doi: 10.1016/j.jlr.2021.100114 (PMC8517605; doi:10.1016/j.jlr.2021.100114)
Supplement: Supplemental data [file mmc1.docx]

**Supplementary Material**

**HSP90 inhibitors reduce cholesterol storage in Niemann-Pick type C1 mutant fibroblasts**

Nina H. Pipalia^1^, Syed Zammam Saad^1^, Kanagaraj Subramanian^2^, Abigail Cross^3^, Aisha al-Motawa^1^, Kunal Garg^1^, Brian S.J. Blagg^4^, Len Neckers^5^, Paul Helquist^4^, Olaf Wiest^4^, Daniel S. Ory^2^ and Frederick R. Maxfield^1,#^

^1^Department of Biochemistry, Weill Cornell Medical College, New York, NY 10065, USA

^2^ Department of Internal Medicine, Washington University in St. Louis, St. Louis MO 63110

^3^ Natural Sciences Department, Fordham University, New York, NY 10023

^4^ Department of Chemistry and Biochemistry, University of Notre Dame, South Bend, IN 46556

^5^ Urologic Oncology Branch, National Cancer Institute, Bethesda, MD 20892

**Methods**

*Statistical Analysis of HSP90 inhibitors dose response plot*

The data for HSP90 inhibitor treated GM18453 NPC1 human skin fibroblasts were analyzed for statistical significance using ANOVA Kruskal-Wallis multiple comparison test in Graphpad PRISM software. The p-values compared to lowest concentration in each treatment are shown in Figure S1

*Co-treatment of Arimoclomol (ACM) with HSF1 Activator (HSF1a)*

Mutant human fibroblasts (GM18453) were purchased from the Coriell Cell Repositories (Camden, NJ) and were grown in Eagle's MEM with Earle's salts and 10% FBS growth media. For screening purposes, growth medium supplemented with 5% FBS was used. We seeded GM18453 cells (450 cells/well in 30 μL) in Corning 384-well black polystyrene flat, clear-bottomed tissue culture-treated plates.

Cells were treated with either ACM alone or in combination with HSF1a in a dose-dependent manner and compared against DMSO-treated controls. The range of drug dosages were made to contain a large gradient of concentrations in screening media as described previously (Pipalia, Huang et al. 2006). Briefly, 15μL of 4x concentration of ACM was combined with either 15μL of 4x concentration of HSF1a and added to appropriate wells to yield 60 µL of 1x concentration of each drug. In control wells corresponding concentrations of DMSO was used.

All plates were incubated with compounds for 72 h at 37°C. Plates were then washed three times with PBS, pH 7.4, using a Bio-Tek Elx405 plate washer (Bio-Tek Instruments, Inc., Winooski, VT). For each wash cycle, 70 μl of PBS was dispensed followed by aspiration with a residual volume of 16 μl/well. Cells were then fixed with 1.5% PFA in PBS for 20 min at room temperature, followed by three more washes with PBS. To the fixed cells, filipin was added at a final concentration of 50 μg/ml in PBS for 45 min at room temperature. Cells were finally washed three times with PBS followed by addition of 2 µM Draq5. Images were acquired three hours after labeling

Measurements were made from four wells for each condition in each experiment, and the experiment was repeated three times. Images were acquired using a 10X dry objective on an ImageXpress^Micro^ fluorescence microscope at two sites per well and analyzed to obtain the LSO compartment ratio. All data were normalized to DMSO treated values.

**Figure. S1.** Dot Plot showing statistical significance of dose dependance of various HSP90 inhibitors using ANOVA Kruskal-Wallis multiple comparison test

1. **B.**

**C. D.**

**E. F.**

**Figure S1. A-F**. Plots showing statistical significance in dose response curve using ANOVA Kruskal-Wallis multiple comparison test in GraphPad PRISM.

**A.** 17AAG, **B**. AUY922, **C.** Ganetespib, **D.** AT13387, **E.** SNX-2112, **F.** TAS116

**Figure S2.**

**Figure S2.**  Individual data points for two EndoH sensitivity assays of NPC1 protein. The fraction of the intensities of EndoH-sensitive (blue open bars and solid circles) and EndoH-resistant (red open bars and solid circles) for each treatment are shown. The methods are presented in the main text.

**Figure S3.**


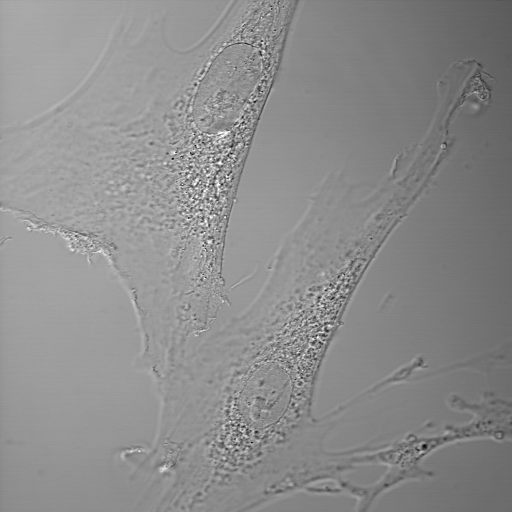

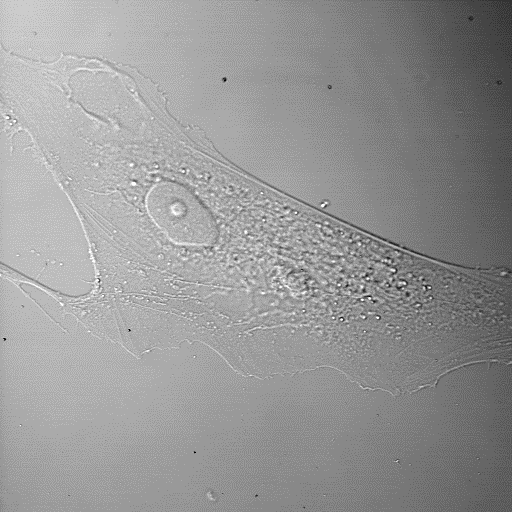

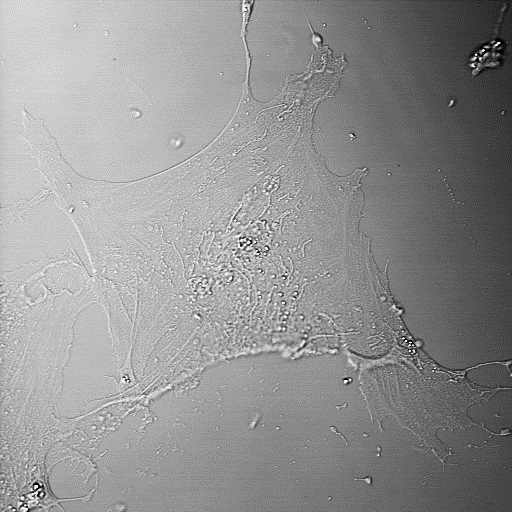


**Transmitted NPC1 LDL Overlay**

**DMSO**

**AUY922**

**(100 nM)**

**AUY922**

**(100 nM)**


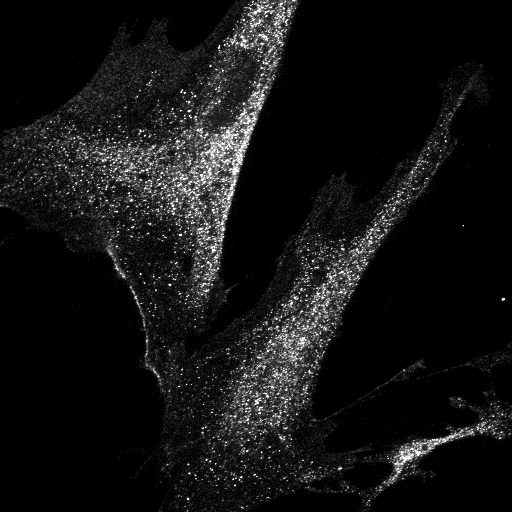

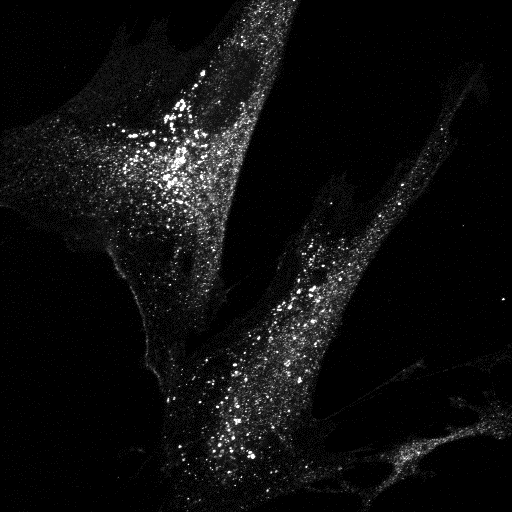

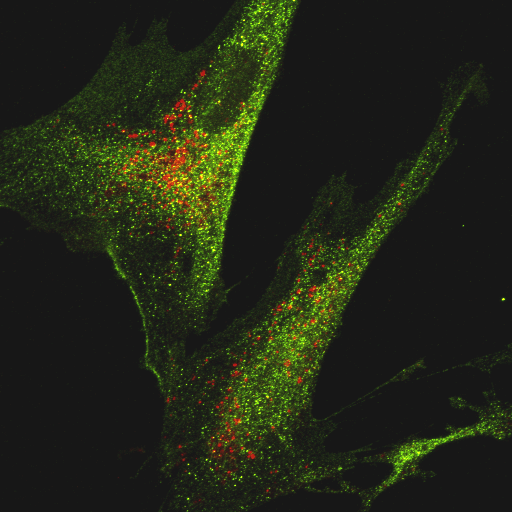

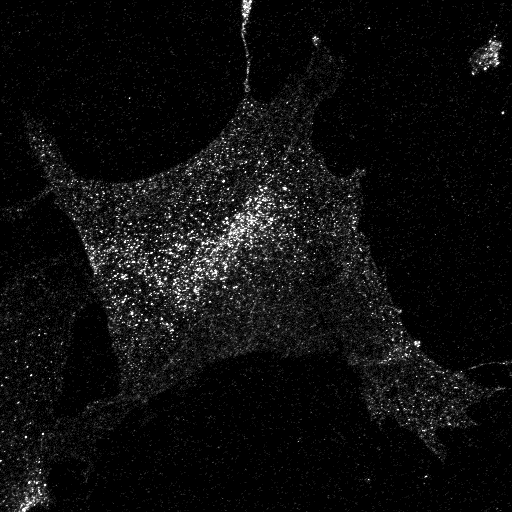

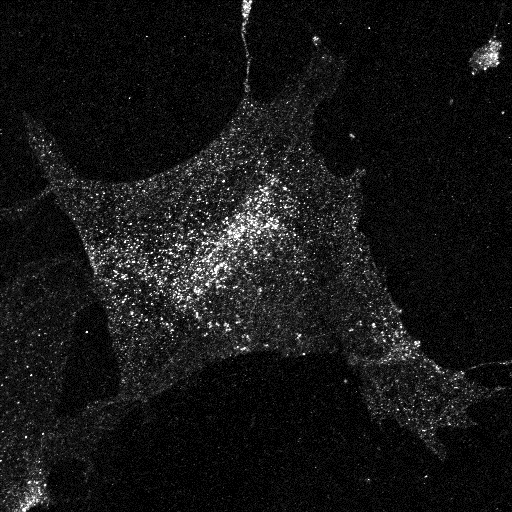

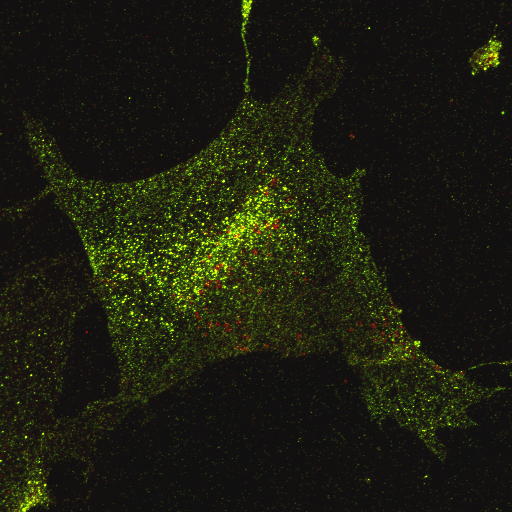

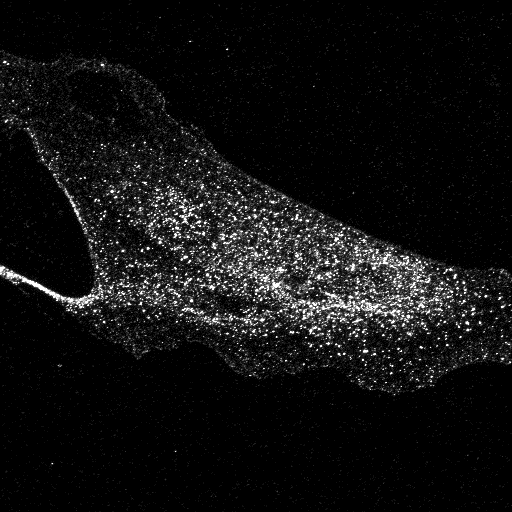

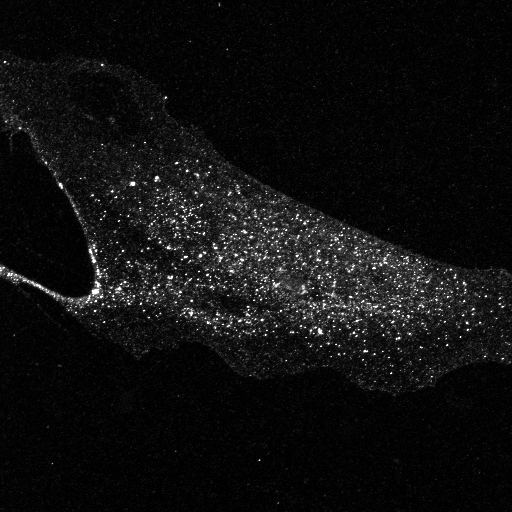

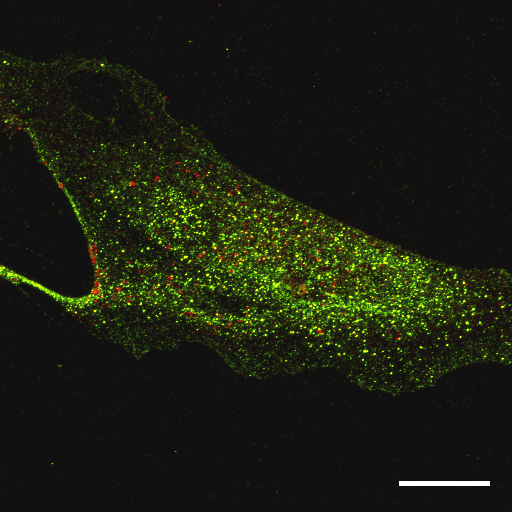


**Figure S3.** GM18453 fibroblasts were treated with 100 nM AUY-922 or solvent control DMSO for 72 h. The cells were incubated with Alexa546 LDL for 3.5 h followed by a 0.5 h chase to deliver labeled LDL to LE/Ly. The fibroblasts were fixed, and NPC1 protein was detected by immunofluorescence. Images were acquired by confocal microscopy. Representative transmitted light and sum projected images of NPC1-A488 and LDL-A546 are shown. Size bar = 25 µm.

**Figure S4.**

1. **B.**

**C. D.**

**Figure S4.** Plot of PFI per GFP+ cell as a function of eGFP-HSP70 and eGFP-HSP40 overexpression.

1. eGFP-Vector, B. eGFP-HSP70, C. eGFP-Vector, D. eGFP-HSP40

**Figure S5.**


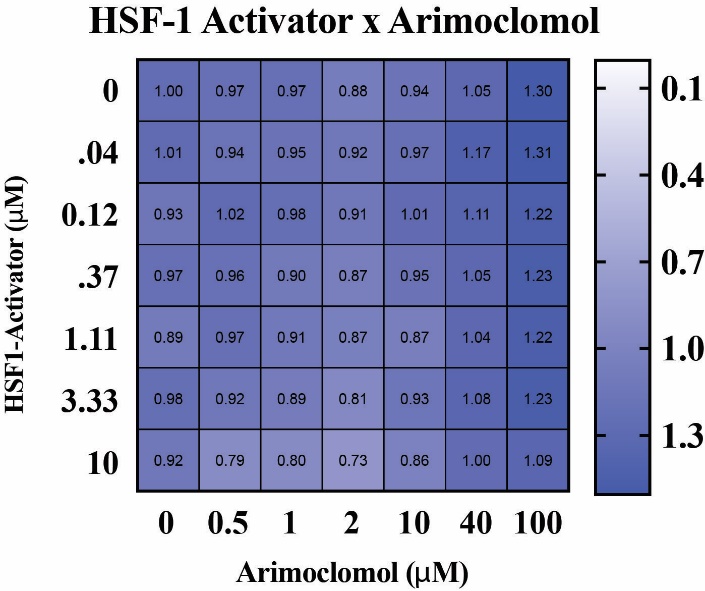


**Figure S5.** *Cotreatment of HSF1a and arimoclomol*

GM18453 fibroblasts were plated in 384-well plates and co-incubated with HSF1a and arimoclomol in a dose-dependent manner for 72 h. Heatmap value represents the average LSO of three independent experiments with four replicates per well per plate, each image had 100-300 cells, normalized to DMSO-treated cells. LSO values less than one represent a decrease in cholesterol storage, and values greater than one identify an increase in cholesterol storage. Concentration on vertical axis represents HSF1a and concentrations on horizontal axis is arimoclomol. All data are statistically insignificant.

**References**

Pipalia, N. H., A. Huang, H. Ralph, M. Rujoi and F. R. Maxfield (2006). "Automated microscopy screening for compounds that partially revert cholesterol accumulation in Niemann-Pick C cells." J Lipid Res **47**(2): 284-301.
